# Repeated colonisation of alpine habitats by *Arabidopsis arenosa* involved parallel adjustments of leaf cuticle traits

**DOI:** 10.1111/nph.70082

**Published:** 2025-03-17

**Authors:** Clara Bertel, Erwann Arc, Magdalena Bohutínská, Dominik Kaplenig, Julian Maindok, Elisa La Regina, Guillaume Wos, Filip Kolář, Karl Hülber, Werner Kofler, Gilbert Neuner, Ilse Kranner

**Affiliations:** ^1^ Department of Botany University of Innsbruck Sternwartestraße 15 Innsbruck 6020 Austria; ^2^ Institute of Ecology and Evolution University of Bern Baltzerstraße 6 Bern 3012 Switzerland; ^3^ Department of Botany Charles University of Prague Benátská 2 Prague 128 01 Czech Republic; ^4^ Polish Academy of Sciences Institute of Nature Conservation al. Adama Mickiewicza 33 Krakow 31‐120 Poland; ^5^ Department of Botany and Biodiversity Research University of Vienna Rennweg 14 Vienna 1030 Austria

**Keywords:** adaptation, Alpine habitat, *Arabidopsis arenosa*, cuticle, cuticular wax composition, ecotype, parallel evolution

## Abstract

- Cuticle function can be pivotal to plant success in different environments. Yet, the occurrence of intraspecific adjustments in cuticle traits resulting from acclimation or adaptation to different habitats remains poorly understood. Here, we used genetically well‐characterised populations of *Arabidopsis arenosa* to investigate whether cuticle traits were adjusted as part of the parallel evolution from a foothill to an alpine ecotype.
- Six alpine and six foothill populations, representing at least three independent evolutionary origins of an alpine ecotype, were used in reciprocal transplantation experiments, to investigate cuticle traits at the eco‐physiological, biochemical and structural levels. The genetic basis behind these traits was assessed by combining selection scans and differential gene expression analysis.
- Overall, alpine populations showed reduced cuticular transpiration in conjunction with consistently altered cuticular wax composition, with higher accumulation of two fatty alcohols and two iso‐alkanes. Genomic analysis unravelled nine genes associated with cuticular wax metabolism showing allelic differentiation in alpine compared to lowland populations. *In silico* gene expression analysis revealed differences between ecotypes for several genes related to cuticle metabolism.
- Repeated ecotypic differentiation in cuticle traits together with the genetic architecture of the alpine ecotype points at an adaptive value of cuticle adjustments for the colonisation of alpine habitats.

## Introduction

When the first plants conquered the land 500 to 450 million years ago (Becker, 2013), several key innovations enabled them to cope with the challenges posed by the new environmental conditions. These challenges included, among other factors, a desiccating atmosphere, higher light intensities and greater temperature fluctuations. One of the innovations was the evolution of the cuticle, an impermeable, highly hydrophobic outermost layer of leaves, young shoots and other aerial parts (Kong *et al*., 2020). The cuticle covers the epidermal cells and acts as a physical barrier against uncontrolled water loss (Burghardt & Riederer, 2006; Kong *et al*., 2020) and also confers protection against various other environmental stress factors. It represents the first barrier to the entry of pests and pathogens (Serrano *et al*., 2014), influences surface properties such as wettability and water run‐off, plays a central role during development by establishing organ boundaries and may be involved in the screening of UV light in some species (Yeats & Rose, 2013). It also helps create a suitable microenvironment for certain microorganisms, the phyllosphere (Kerstiens, 2006; Riederer, 2006). Accordingly, cuticle traits can vary within a plant species, depending on the habitat (Xue *et al*., 2017).

Plant cuticles are composed of two highly hydrophobic components, cutin and cuticular waxes, which are assembled in several layers. While cutin mainly provides mechanical strength, cuticular waxes determine water permeability, leaf wettability and light reflectance, and thus play important roles in the adaptation to environmental stress factors, including drought, temperature fluctuations and plant–pathogen interactions. Cuticular waxes are embedded within the cuticle and also deposited as crystals on the surface (Bernard & Joubes, 2013). Key functional traits of cuticles, especially cuticle permeability for water vapour, are not directly related to cuticle thickness or to the total amount of waxes, but rather to the composition of its layers, and specifically to the accumulation of very‐long‐chain aliphatics (VLCA) (Jetter & Riederer, 2016; Seufert *et al*., 2022). The main classes of VLCAs found in cuticular waxes are alkanes, aldehydes, primary and secondary alcohols, ketones and esters (Yeats & Rose, 2013), which may contribute to reducing transpiration to varying degrees (Grncarevic & Radler, 1967). For instance, the water vapour permeability determined for films of pure compounds was lower for aldehydes and very long chain alcohols than for alkanes of similar carbon chain length (Leyva‐Gutierrez & Wang, 2021). Pathways for the biosynthesis of cutin and cuticular waxes have been partially characterised in *Arabidopsis thaliana* (Bernard & Joubes, 2013; Philippe *et al*., 2020), the differential regulation of which may affect cuticle function, in particular the control of water loss (Asadyar *et al*., 2024).

One measure of water deficiency in leaves is the water saturation deficit (WSD), which represents the proportion of water missing to reach full saturation, reflecting how well the cuticle protects the leaf tissue from water loss after stomatal closure (Larcher, 2001). To reduce water loss, higher plants close their stomata. However, they still transpire through the cuticle and incompletely closed stomata (Schuster *et al*., 2017; Duursma *et al*., 2019), together constituting the leaf minimum conductance (*g*_min_). This trait varies greatly among species, with some phenotypic plasticity, and is adjusted in relation to the micro‐climate, especially by abiotic factors that can influence the evaporative demand such as wind, drought and temperature (Fernández *et al*., 2017; Duursma *et al*., 2019; Körner, 2021). Furthermore, foliar water balance is influenced by leaf surface wettability, that is, how easily a liquid such as water spreads over the leaf surface (Barthlott & Neinhuis, 1997), which is affected by the abundance, nature and density of trichomes (Brewer *et al*., 1991). Leaf wettability decreases with increasing elevation in relation to both changing species composition and intraspecific adjustments (Aryal & Neuner, 2010), and confers important leaf functions, affecting both water uptake through leaf surfaces and transpiration (Goldsmith *et al*., 2017). However, surface water can also reduce gas exchange and carbon assimilation (Brewer & Smith, 1995; Ishibashi & Terashima, 1995), promote pathogen growth (Evans *et al*., 1992), lead to leaching of leaf nutrients, and can increase biomechanical stress (Cape, 1996). In addition, the probability of extrinsic ice nucleation increases with the amount of water retained on leaves (Wisniewski *et al*., 2002), hence an increased leaf water repellency may be beneficial in alpine environments (Yumoto *et al*., 2021). In summary, environmental conditions during plant growth and development apparently influence cuticle composition, *g*_min_, and leaf wettability. However, in spite of the known importance of cuticle traits for plant adaptation to the external environment (González‐Valenzuela *et al*., 2023), the extent to which the interplay between the environmental factors and intraspecific genetic variation, possibly resulting from evolutionary adjustments, shapes these leaf traits remains poorly understood.

Naturally replicated cases of evolution encompassing parallel evolution of specific traits provide an opportunity to distinguish adaptive traits from neutral changes (Bolnick *et al*., 2018; James *et al*., 2023). In *Arabidopsis arenosa*, the repeated emergence of an alpine *A. arenosa* ecotype from a broadly distributed foothill ecotype, in at least three European mountain ranges, was accompanied by parallel changes in multiple morphological, anatomical and functional traits such as petal size, plant height (Knotek *et al*., 2020), leaf thickness, trichome density (Bertel *et al*., 2022) and cold acclimation potential (Kaplenig *et al*., 2022). This congruent ecotypic differentiation has been associated with a higher fitness of populations in their local vs foreign habitats (Wos *et al*., 2022). Aiming to uncover the genomic and transcriptomic basis of parallel adaptation to the alpine habitat in *A. arenosa*, previous studies identified 151 candidate genes that were repeatedly differentiated between foothill and alpine populations (Bohutínská *et al*., 2021) and these changes likely led to broad, partially parallel, transcriptomic responses (Wos *et al*., 2021). However, the extent to which genetic changes affected cuticle synthesis pathways, potentially affecting cuticle composition, *g*_min_ and leaf wettability, remains unknown.

Leveraging the established case of parallel evolution in *A. arenosa*, we explored the relevance of cuticle traits in the adaptation to the alpine habitat, accounting for ecotypic differentiation based on detailed knowledge of population genetic differentiation (Knotek *et al*., 2020). We used six foothill and six alpine populations, representing three independent evolutionary origins of an alpine ecotype, for which other phenotypic traits have already been linked to a fitness advantage in their local habitat (Wos *et al*., 2022). In line with previous studies, we used a reciprocal transplantation experiment at elevations where the alpine and foothill *A. arenosa* ecotypes occur, to test whether alpine populations show heritable and consistent changes in cuticle eco‐physiological traits compared to foothill ones. We also compared the composition of cuticular waxes to determine whether biochemical changes could explain the ecotypic differentiation. Furthermore, to elucidate the genetic basis of cuticle trait differentiation in *A. arenosa*, we re‐analysed available genomic data (Bohutínská *et al*., 2021, 2023; Wos *et al*., 2021), combining selection scans and differential gene expression analysis between foothill and alpine populations, focusing on a set of known cuticle‐related genes.

## Materials and Methods

### Plant material and growth

Six alpine and six foothill autotetraploid *Arabidopsis arenosa* (L.) Lawalrée populations from three European mountain ranges, the Niedere Tauern (Eastern Alps, Austria, NT), the Tatra Mountains (TM) and the Făgăraș Mountains (Southern Carpathians, Romania, FG), were studied (Supporting Information Fig. S1). Seeds were collected in the field and regenerated under controlled conditions as previously described (Knotek *et al*., 2020; Wos *et al*., 2021; Bertel *et al*., 2022; Kaplenig *et al*., 2022). Note that the same populations were used in previous studies, focusing on local adaptation (Wos *et al*., 2022), freezing tolerance (Kaplenig *et al*., 2022) and leaf anatomy (Bertel *et al*., 2022), but mostly in separate reciprocal transplantation experiments. See Knotek *et al*. (2020) for details on the morphological phenotype of the plants and the collection sites of the populations studied (listed in Table S1).

Cuticle traits were studied in fully developed and healthy rosette leaves of plants grown in a reciprocal transplantation experiment in two common gardens at different elevations, as previously described (Bertel *et al*., 2022). The ‘foothill common garden’ was located in the Botanical Garden of the University of Innsbruck, Tyrol, at 600 m asl (47°16′04.7″N 11°22′47.9″E) and the ‘alpine common garden’ in the Alpine Garden of the University of Innsbruck on Mt. Patscherkofel at 1960 m asl (47°12′38.8″N 11°27′05.7″E). Leaf cuticle wax composition and surface structure were assessed only of plants grown in the alpine common garden. See Methods S1 for further details.

### Leaf minimum conductance and wettability

Leaf minimum conductance (*g*_min_) was derived from leaf drying curves (Cape & Percy, 1996). Potted plants were removed from the common gardens, watered and left overnight in a dark room to achieve full hydration. Immediately after leaf detachment, the saturation mass (SM) of the rosette leaves was determined using a precision balance (Quintix 65‐1s; Sartorius, Göttingen, Germany) and a scaled image of the leaves was taken (Canon EOF 70D) to determine leaf area (*A*). Leaves were then air‐dried at room temperature under low irradiance (< 2 μmol photons m^−2^ s^−1^) on a wire mesh to ensure free air circulation and unimpeded transpiration. Leaf fresh mass (FM) was determined at 30 min intervals for a period of 150 min and the corresponding mass losses (∆*M* = SM – FM) were calculated. Then, leaves were dried in an oven (T 6060; Heraeus, Hanau, Germany) for 48 h at 80°C and reweighed to determine the dry mass (DM). The transpiration rate (*J*_H20_) was determined for each time interval (∆*t*) as $J_{H_{2}0}=\frac{\Delta M}{A*\Delta t}$. After stomatal closure, *J*_H20_ levelled off to a minimum value and after 90 min of air drying, *J*_H20_ remained constantly low. Therefore, the values measured after 90 min of leaf drying were used for further calculations, and *g*_min_ was calculated as *g*_min_ = $J_{H_{2}0}$/∆$C_{H_{2}0}$ (Larcher, 2001), where ∆$C_{H_{2}0}$ is the difference between the water vapour concentration outside the leaf (*c*_a_) and that in the intracellular spaces (*c*_i_). *c*_a_ was determined from the relative humidity, ambient temperature and air pressure (Willert *et al*., 1995), recorded after each weighing using a hygro‐thermo‐barometer (GFTB 200, Greisinger Messtechnik GmbH, Regenstauf, Germany). *c*_i_ was determined accordingly, assuming 100% relative humidity in the leaf intracellular space. In addition, the water saturation deficit (WSD) was calculated as $\mathrm{WSD}=\frac{\Delta M}{\mathrm{SM}-\mathrm{DM}}\times 100$. *g*_min_ and WSD was determined for 14 leaves per population, each sampled from a randomly selected, individual plant (Dataset S1).

To assess leaf wettability, leaves were detached and immediately taped to a flat surface with double‐sided tape (Aryal & Neuner, 2010). Then, a 5 μl drop of tap water was placed on the leaf surface using a microlitre syringe (Hamilton Bonaduz AG P.O., Bonaduz, Switzerland). The contact angle was determined from digital images of droplets on leaves photographed in side view (Apple iPhone 8 camera) using imagej (National Institutes of Health, Bethesda, MD, USA) with the open‐source plug‐in ‘Contact Angle’ (https://imagej.nih.gov/ij/plugins/contact‐angle.html, last accessed 19 November 2019) for both the adaxial (upper) and abaxial (lower) sides of the leaves. The contact angle (θ) was determined by a line tangent and the contact points of the droplet on the leaf surface were measured. The smaller θ, the more wettable is a leaf. θ was measured on 14 leaves per population, each sampled from a randomly selected, individual plant (Dataset S1).

### Leaf surface structure

To visualise surface structures by scanning electron microscopy, leaves were sampled from plants grown in the alpine common garden. Potted plants were transferred to the Botanical Garden Innsbruck and leaves were sampled in 5 ml Eppendorf tubes and processed immediately to avoid structural degradation by evaporation. To dehydrate the leaves slowly, 4 ml of increasing concentrations of ethanol (10, 20, 30, 40, 50, 75 and 96%) was added sequentially to each tube. Each dehydration step was carried out for *c*. 60 min, after which the previously used lower concentration solution was discarded and replaced by a solution of the next higher concentration. The last step, using 96% ethanol, was repeated three times. The tubes were stored in the dark at 4°C until further chemical fixation. Dehydrated leaves were incubated in concentrated formaldehyde dimethyl acetal overnight and subjected to critical‐point‐drying using liquid CO_2_ at a temperature of 31°C and a pressure of 73.8 bar (CPD 30, Balzers, Liechtenstein). Sample material was stored in a desiccator over silica gel until further preparation. Leaves were cut into fragments using tweezers and a scalpel, which were placed on adhesive foil over aluminium stubs (Zeiss), and examined under a stereomicroscope (M3Z, Wild, Heerbrugg, Switzerland). To increase the electrical conductivity of the sample material, two to five surface points of each fragment were connected to the metal stand with graphite (Leit‐C nach Göcke). The aluminium stubs supporting the sample material were placed in a sputtering system (EM SCD050; Leica, Wetzlar, Germany) to coat the surface with a gold layer (25 nm) using an argon atmosphere at a working distance of 50 mm, a pressure of 0.05 mbar, a sputtering time of 260 s and a sputtering current of 250 V 15 mA^−1^. The sputtered sample material was stored overnight in an oven at 40°C until further examination under the scanning electron microscope (EVO 10; Zeiss).

### Cuticle wax analyses

For each population, one to three healthy rosette leaves, each from eight to nine randomly selected individual plants, were sampled between 9:00 h and 10:00 h and pooled. The number of leaves sampled per individual was visually adjusted to compensate for differences in leaf size and to obtain comparable total leaf areas per individual and per population (216.3 ± 43.9 mm^2^). Sampling was repeated on four consecutive days, with leaves collected from different subsets of plants every day. Sampled leaves were immediately placed in‐between two water‐dampened filter papers (Whatman Grade 1) and transported back to the laboratory within the next hour. Leaves were photographed using a digital camera (Sony DSC‐RX100III; Sony Europe, Weybridge, UK) and leaf surface area was determined using imagej (total leaf area per sample was *c*. 2.16 ± 0.44 cm^2^). Cuticular waxes were extracted and analysed using a protocol adapted from Yumoto *et al*. (2021). For each sample, waxes were extracted by immersing the pooled leaves, placed in a 15‐ml glass tube together with 10 μl of 1 mM tetracosane as internal standard, in 3 ml of chloroform for a total of 30 s at room temperature, including 15 s of gentle vortexing. The extracts were collected in a new 15‐ml glass tube and the solvent was completely evaporated under a gentle stream of nitrogen gas at 30°C using a 6‐port Mini‐Vap evaporator/concentrator (Merk, Darmstadt, Germany). Waxes were resuspended and derivatised by adding 75 μl of pyridine and 75 μl of N‐Methyl‐N‐trimethylsilyl‐trifluoroacetamide (MSTFA), followed by 1 h of incubation at 80°C.

A 1‐μl aliquot of the resulting sample was injected into the split‐splitless inlet of a Trace 1300 gas chromatograph (Thermo Scientific, Waltham, MA, USA), operated at 280°C in split mode with a carrier flow of helium at 1 ml min^−1^ and a split flow of 20 ml min^−1^. Analytes were separated on a 30‐m Rxi‐5Sil MS column with a 10‐m Integra‐Guard pre‐column (Restek, Bellefonte, PA, USA) using an oven temperature ramp starting at 70°C for 7 min, followed by an increase of 10°C min^−1^ up to 325°C, which was held for 10 min. Compounds were detected using a TSQ8000 triple quadrupole mass spectrometer (Thermo Scientific) operated in full scan mode, scanning from 50 to 550 *m*/*z* after 9 min solvent delay, with the transfer line and ion source temperatures set at 300 and 330°C, respectively. An alkane mixture was injected in the middle of the queue for external retention index calibration. The Xcalibur software (v.4.2; Thermo Scientific) was used for data acquisition in combination with the NIST, Golm and Fiehn mass spectral libraries for compound identification. Peak areas for compound‐specific ions were used for relative quantification of identified metabolites (Dataset S1).

### Statistical analysis

Data were analysed for significance at *P* < 0.05 using R (R Core Team, 2024). To test for differences in *g*_min_, WSD and leaf wettability, linear mixed‐effects models (LMEs), as implemented in the function ‘lmer’ in the package ‘lmertest’ (Kuznetsova *et al*., 2017), were applied separately for each trait. To test for ecotypic differentiation and plastic adjustment in traits between tetraploid foothill and alpine populations, and parallel evolution of the latter in the three mountain ranges, ecotype (foothill vs alpine), common garden (foothill vs alpine) and mountain range, and the interactions ecotype–common garden and ecotype–mountain range were regressed as fixed effects on each trait. Source population was used as random intercept to account for the potential non‐independence of values derived from seeds sampled within the same population. Parameters were estimated by optimising the restricted maximum likelihood criterion. Model assumptions of normality and homogeneity of error variance were checked by diagnostic plots, calculating variance inflation factors and testing for homogeneity of variance by Levene's test. Differentiation in the accumulation of individual cuticle wax compounds was analysed using LMEs, with the same model structure as described above except that common garden was omitted as fixed factor, applied separately for each compound. Before model fitting, traits were transformed by natural logarithm (*g*_min_, cuticular waxes) or square root (leaf wettability, WSD). Multiple testing was corrected for by Benjamini–Hochberg correction (Benjamini & Hochberg, 1995).

### Targeted re‐analysis of genomic and transcriptomic data

Genome‐wide scans for footprints of natural selection were conducted using whole‐genome re‐sequencing data generated from 420 individuals of *A. arenosa* (Novikova *et al*., 2016; Monnahan *et al*., 2019; Preite *et al*., 2019; Bohutínská *et al*., 2021; Konečná *et al*., 2021, compiled in Bohutínská *et al*., 2023) collected by range‐wide sampling and including alpine populations occurring between 1600 and 2500 asl and foothill populations between 400 and 900 asl (Table S2). Of note, this set included all 12 populations used in this study. For this dataset, whole genomes were processed and filtered as previously described (Monnahan *et al*., 2019). Final variant call format (VCF) files were annotated using SNPeff and variants spanning either the coding region or the *cis*‐regulatory regions (5 kb upstream and downstream) of any of a set of 104 genes, comprising all cuticle‐related genes identified in previous studies in *A. thaliana* (Yeats & Rose, 2013; Philippe *et al*., 2020) and from the TAIR database (Bernard *et al*., 2012) were extracted (Table S3). For the selection scans, the allele frequency difference (AFD) was used as a measure of genetic differentiation (Bertel *et al*., 2018). The selection scan was performed using the NatGenVarViewer R script (github.com/mbohutinska/NatGenVarViewer). Briefly, genes were scanned for outlier single nucleotide polymorphisms (SNPs) discriminating between foothill and alpine individuals of all mountain regions with a 0.3% outlier AFD cut‐off and the density of outlier SNPs per gene was calculated using *A. lyrata* gene models (Rawat *et al*., 2015). We identified 50% of cuticle genes with the highest outlier SNP density as candidate genes (Table S4). We selected a more stringent AFD cut‐off than the usual 1% or 5%, as our analysis focused specifically on cuticle genes where we expected to see evidence of selection, rather than across the entire genome where more neutrally evolving sites might be expected. This approach, along with filtering the top half of genes with more candidate SNPs, aimed to reduce false positives, a common issue in selection scans (Mallick *et al*., 2009). Candidate gene identification was facilitated by the high nucleotide diversity present in this outcrossing species (Yant & Bomblies, 2017).

To investigate the differential expression of cuticle‐related genes, we re‐analysed a previously generated RNA‐seq dataset covering both ecotypes and all regions (Wos *et al*., 2021). Briefly, the design involved seedlings from one alpine and one foothill population per region raised in growth chambers under conditions varying in temperature and irradiance, two variables strongly associated with elevation gradients. RNA sequencing was performed on non‐flowering plants at the 14‐leaf stage (for details of the design and selected values for temperature and irradiance, see Wos *et al*., 2021). The edger package was used to test for consistent differential gene expression between all foothill and all alpine individuals (i.e. the parallel differential gene expression), irrespective of their growth conditions (i.e. the gene expression likely to be determined by genetic rather than plastic response). Briefly, library sizes (i.e. read counts) were scaled and normalised, dispersion estimated and the ‘glmFit’ function used to test for gene expression differences between alpine and foothill populations, with growth condition as a covariate. Genes, with read counts > 1000, were considered as differentially expressed if FDR < 0.1 (Table S5).

## Results

### Ecotypic differentiation in cuticle‐related leaf traits

**Fig. 1:**
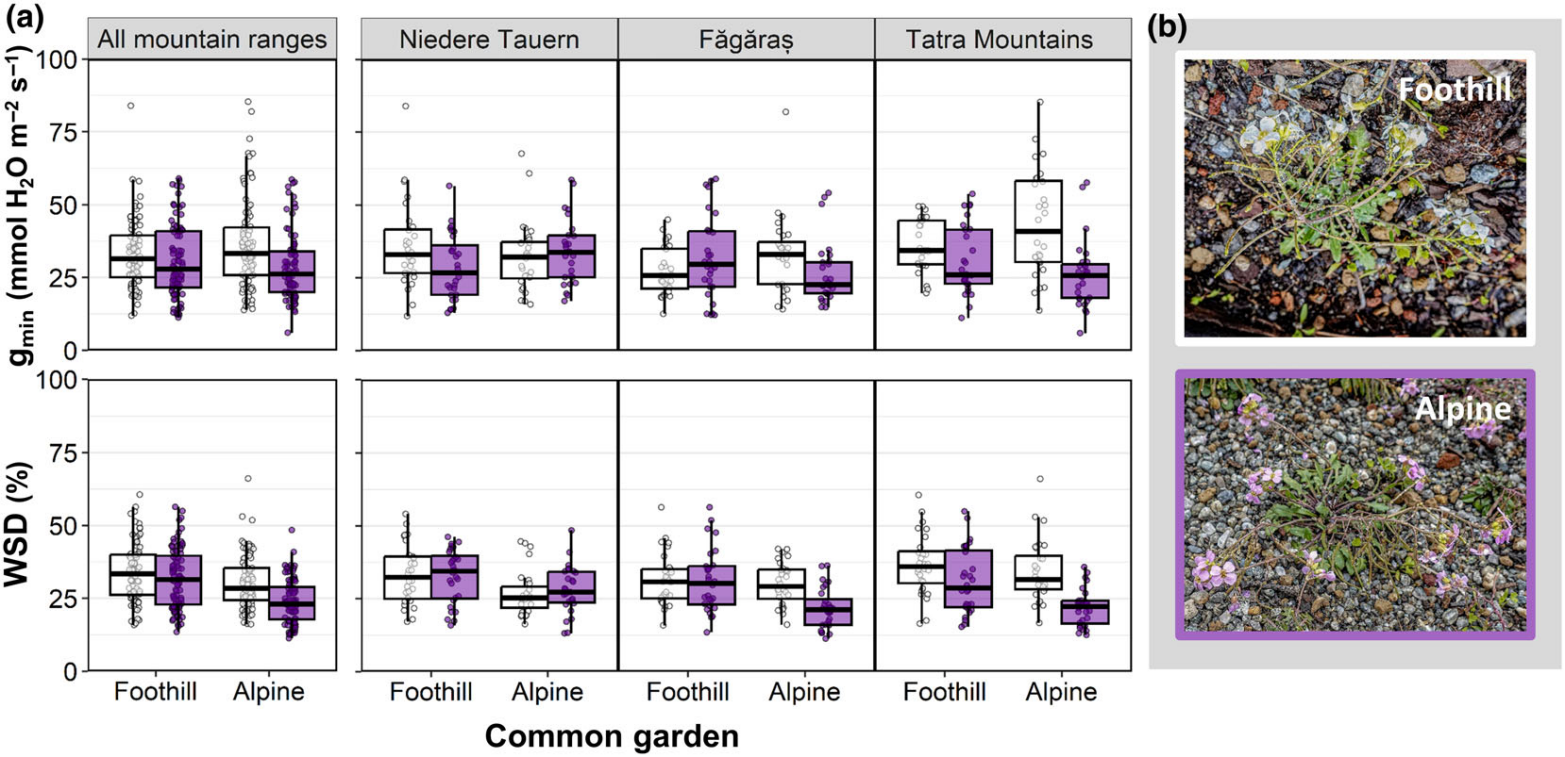
Minimum leaf conductance (*g*_min_) and water saturation deficit (WSD) of foothill (white) and alpine (purple) populations of *Arabidopsis arenosa*, grown in a foothill and an alpine common garden. (a) *g*_min_ and WSD values for tetraploid populations shown pooled together for all three mountain ranges and separately for each mountain range. Boxes depict the interquartile range (25^th^–75^th^ percentiles), with the median shown as a horizontal line, whiskers extend to data points (dots) within 1.5 time the interquartile range. Individual data points beyond this range are outliers. (b) Individuals from the foothill (top; white flowers) and alpine (bottom; purple flowers) *A. arenosa* ecotypes grown in a common garden, in the Alpine Garden of the University of Innsbruck on Mt. Patscherkofel.

Across all the populations studied, *g*_min_ differed between the two ecotypes (Table 1). The alpine ecotype generally showed lower *g*_min_ values, especially when grown in the alpine common garden (Fig. 1), indicating higher water retention capacity. When grown in the alpine common garden, populations from the FG and TM regions showed the most pronounced differences, with overall lower *g*_min_ values in the alpine than in the foothill populations (Fig. 1). By contrast, neither the mountain range, from which the populations originated, nor the common gardens, where plants were grown, significantly influenced leaf *g*_min_ (Table 1).

**Table 1:** Type III analysis of variance table obtained from linear mixed‐effects models relating ecotype, common garden and mountain range of origin to cuticle traits of *Arabidopsis arenosa* populations from three mountain ranges.

	Sum of squares	df	*F* value	*p‐*value
**Leaf minimum conductance (*g***_**min**_ **)**				
Ecotype	0.979	1	6.306	**0.046**.
Common garden	0.031	1	0.197	0.658
Mountain range	0.469	2	1.512	0.294
Ecotype: common garden	0.217	1	1.395	0.238
Ecotype: mountain range	0.741	2	2.388	0.172
**Water saturation deficit (WSD)**				
Ecotype	5.257	1	7.915	**0.031**
Common garden	20.906	1	31.481	**< 0.001**
Mountain range	0.925	2	0.697	0.535
Ecotype: common garden	3.777	1	5.687	**0.018**
Ecotype: mountain range	3.910	2	2.943	0.129
**Leaf wettability, adaxial**				
Ecotype	1.521	1	1.966	0.209
Common garden	43.434	1	56.131	**< 0.001**
Mountain range	2.137	2	1.381	0.320
Ecotype: common garden	0.099	1	0.128	0.721
Ecotype: mountain range	2.246	2	1.451	0.305
**Leaf wettability, abaxial**				
Ecotype	0.597	1	0.498	0.504
Common garden	174.077	1	145.343	**< 0.001**
Mountain range	21.224	2	8.860	**0.015**
Ecotype: common garden	0.651	1	0.544	0.461
Ecotype: mountain range	17.502	2	7.306	**0.023**

Degrees of freedom (df) were approximated using Satterthwaite's method (Kuznetsova *et al*., 2017). Nesting of populations within mountain ranges was accounted for by introducing a random intercept in the model. *P*‐values below 0.05 are displayed in bold.

Water saturation deficit differed according to ecotype and common garden (Table 1). In general, plants grown in the alpine common garden had a lower WSD than those grown in the foothill common garden (Fig. 1). Within the alpine common garden, the alpine ecotype had a lower WSD than the foothill ecotype in populations originating from FG and TM (Fig. 1), providing further evidence that the alpine ecotype can adjust its leaf characteristics to achieve a higher water retention than the foothill ecotype.

**Fig. 2:**
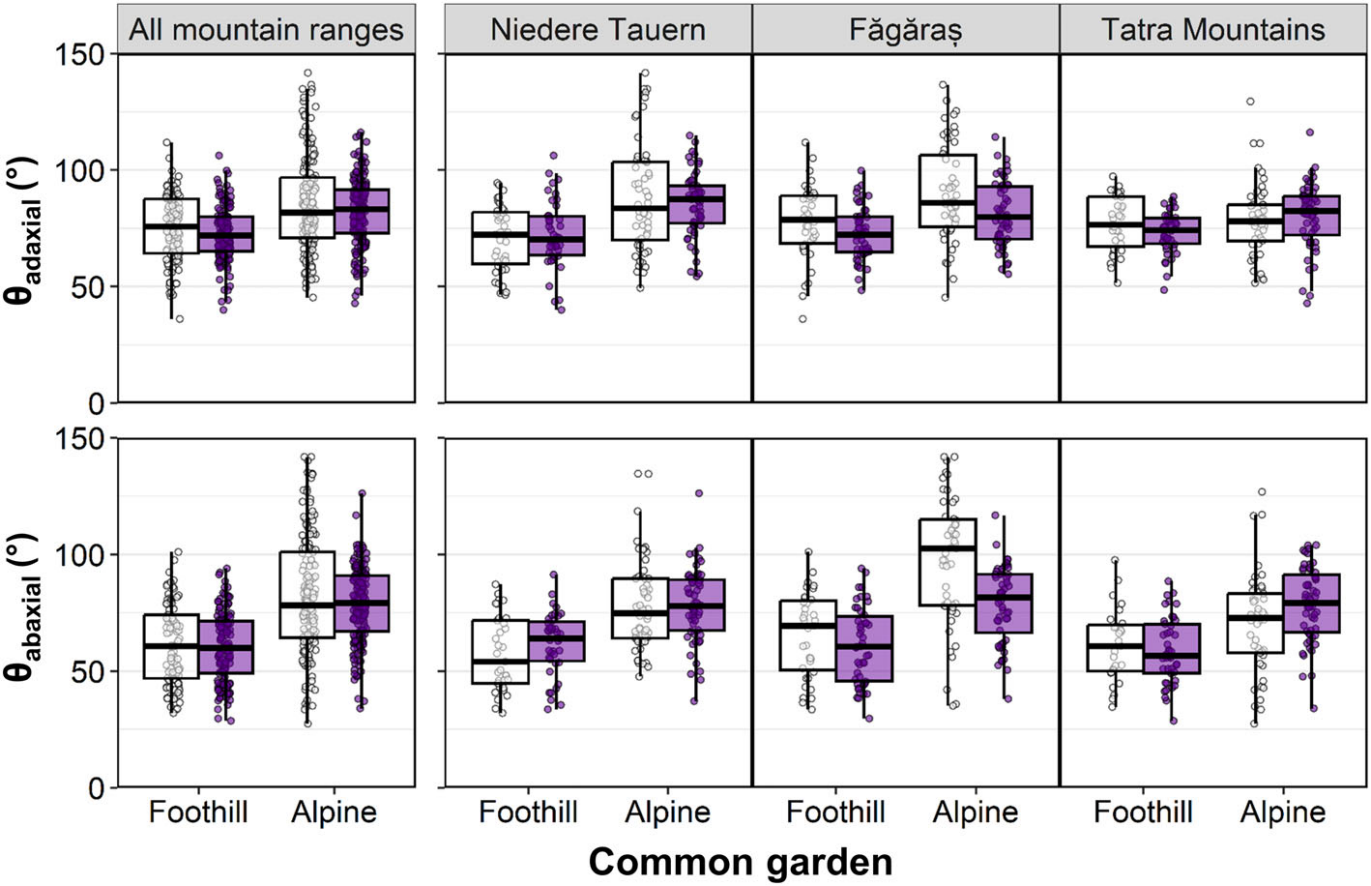
Leaf wettability of adaxial and abaxial leaf surfaces of foothill (white) and alpine (purple) tetraploid populations of *Arabidopsis arenosa*, grown in a foothill and an alpine common garden. Wettability was assessed by measuring the contact angle (θ), that is the angle between the leaf surface and the tangent to a water droplet at the point where air, leaf and water meet. The droplet contact angle is inversely related to leaf wettability. Results are shown for all populations pooled for the three mountain ranges and separately for each mountain range. Boxes depict the interquartile range (25^th^–75^th^ percentiles), with the median shown as a horizontal line, whiskers extend to data points (dots) within 1.5 time the interquartile range. Individual data points beyond this range are outliers.

The wettability of both adaxial and abaxial leaf surfaces differed between the two common gardens, with lower wettability observed for plants grown in the alpine common garden, but did not vary consistently between ecotypes (Table 1; Fig. 2). However, a significant interaction between the factors ecotype and mountain range was observed for the wettability of the abaxial leaf surface, but not for the adaxial leaf surface (Table 1). Furthermore, no visual differences in leaf surface structures were observed between ecotypes by scanning electron microscopy (Fig. S2).

### Ecotypic differentiation in cuticular wax composition

**Fig. 3:**
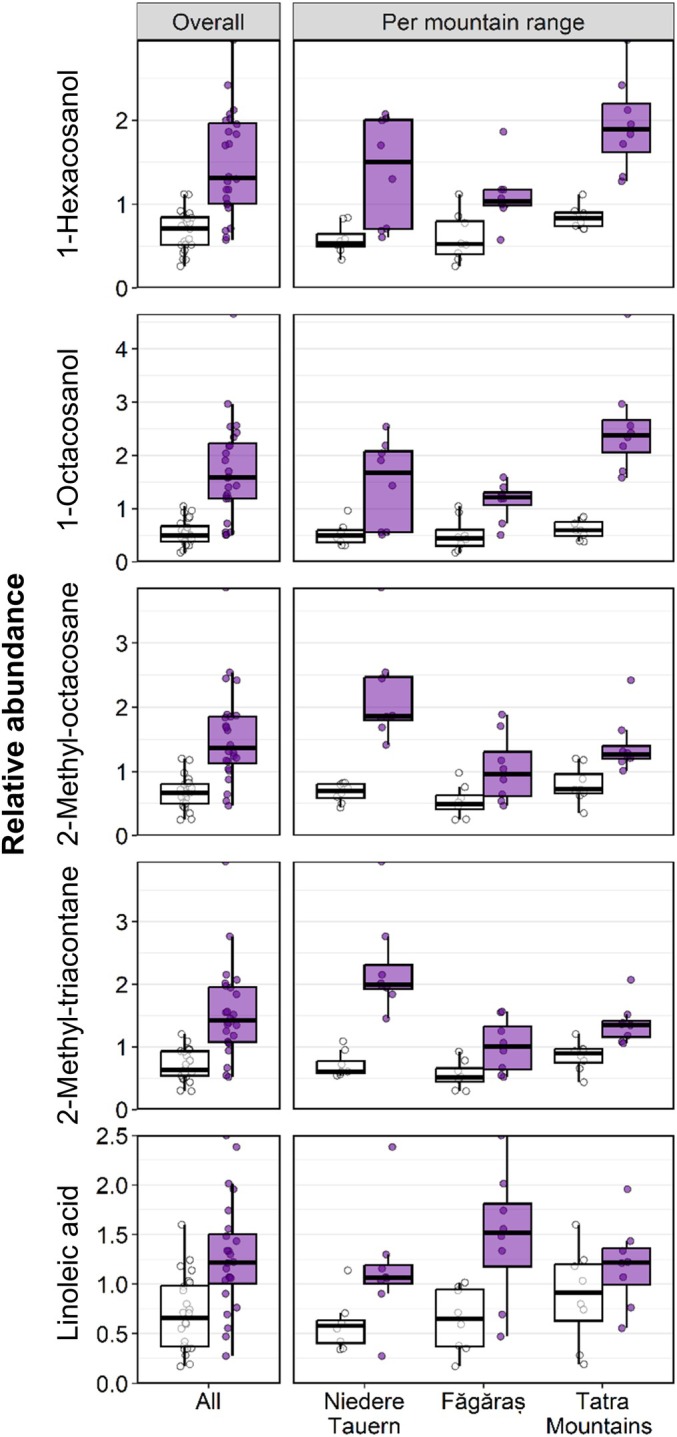
Ecotypic differentiation in cuticular wax composition of leaves from tetraploid *Arabidopsis arenosa* populations. Cuticular waxes were extracted from leaves of alpine and foothill populations grown in an alpine common garden and analysed by GC‐MS. Of the 29 wax compounds identified, only those that were found to accumulate differentially between the foothill (white) and alpine (purple) ecotypes are shown (statistics in Supporting Information Table S6). Boxes depict the interquartile range (25^th^–75^th^ percentiles), with the median shown as a horizontal line, whiskers extend to data points (dots) within 1.5 time the interquartile range. Individual data points beyond this range are outliers.

The main compounds identified in the leaf cuticular waxes were alkanes, namely nonacosane (C29), hentriacontane (C31) and tritriacontane (C33), fatty alcohols, including 1‐hexacosanol (C26‐OH) and 1‐octacosanol (C28‐OH), and long chain fatty acids. Although the same compounds were detected in the waxes of all populations, the alpine and foothill ecotypes consistently differed in the accumulation of the two most abundant primary alcohols (1‐hexacosanol and 1‐octacosanol), of two branched alkanes and of linoleic acid (Fig. 3; Table S6). Leaves from alpine populations showed at least twice the surface concentration of these molecules compared to those from foothill populations. By contrast, the mountain range, from which the populations originated, did not significantly influence the accumulation of any compound (Table S6).

### Genetic and transcriptomic differentiation in cuticle‐related genes

**Fig. 4:**
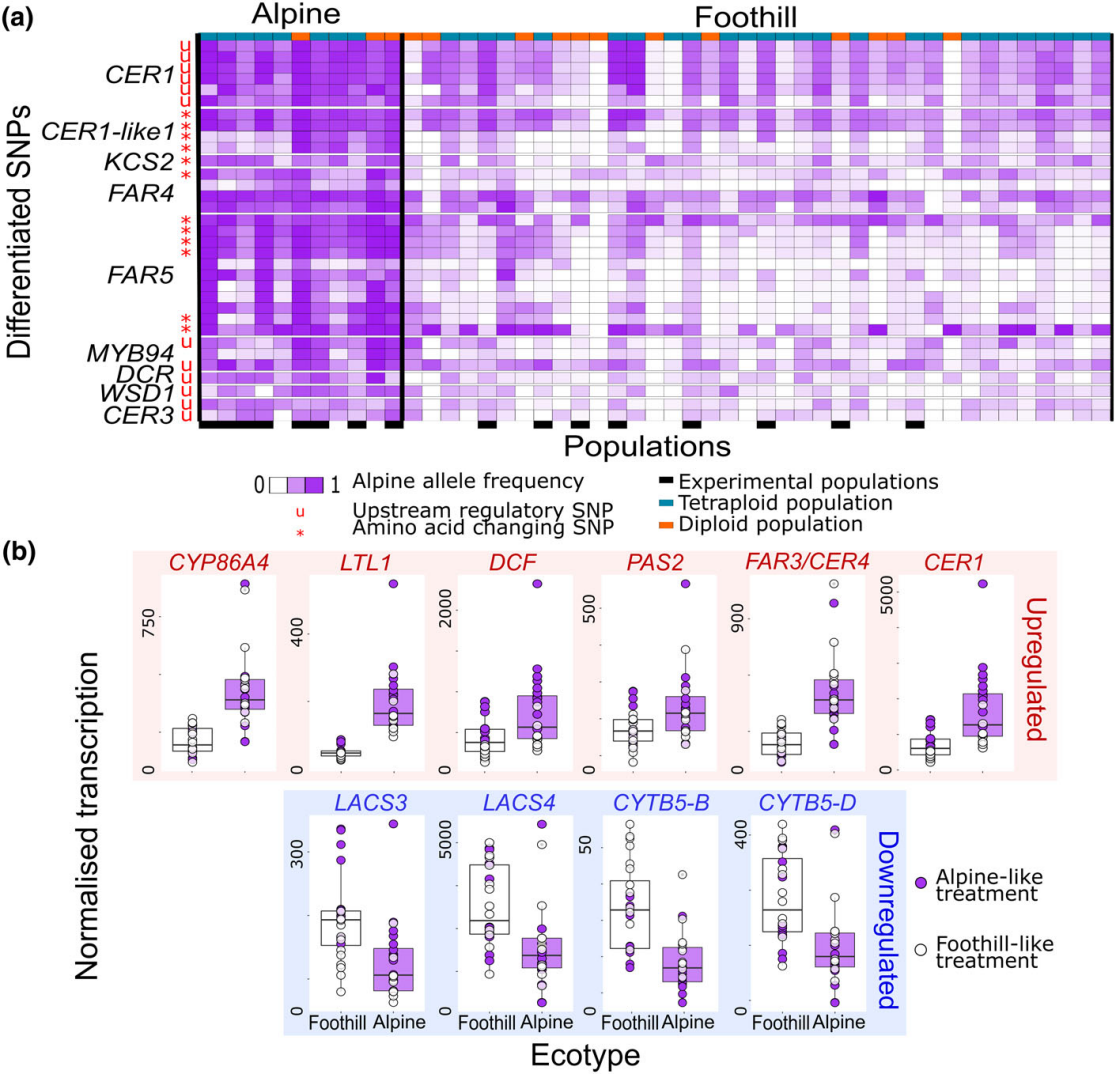
Genetic and transcriptomic differences in cuticle‐related genes associated with the alpine environment in *Arabidopsis arenosa*. (a) Thirty‐four single nucleotide polymorphisms (SNPs; rows) found in nine cuticle‐related genes (shown on the left‐hand side) showed outlier differentiation between alpine and foothill populations. The colour ramp from white to purple indicates the frequency of alpine alleles; red letters on the left‐hand side indicate upstream regulatory SNPs and stars show amino acid‐changing SNPs. (b) In alpine populations, six and four genes were significantly (FDR < 0.1) up‐ and down‐regulated, respectively, independent of the growth conditions. Data were re‐analysed from Wos *et al*. (2021), who grew *A. arenosa* populations under controlled conditions reflecting the contrasting light and temperature conditions experienced by the plants in the foothill (white dots) and alpine habitats (purple dots). Boxes depict the interquartile range (25^th^–75^th^ percentiles), with the median shown as a horizontal line, whiskers extend to data points (dots) within 1.5 time the interquartile range. Individual data points beyond this range are outliers.

Selection scans over a predefined set of cuticle‐related genes (Table S3) led to the identification of a set of 34 SNPs that showed significant allele frequency differentiation between foothill and alpine populations of all mountain ranges (Table S4), overlapping with either the upstream regulatory or the coding region of nine cuticle genes (Fig. 4a). These genes included *ECERIFERUM 1* (*CER1*), *ECERIFERUM 3* (*CER3*) and *ECERIFERUM 1‐like 1* (*CER1‐like1*), which are involved in the conversion of acyl‐coenzyme A to alkanes; *FAR4* and *FAR5*, which encode fatty acyl‐coenzyme A reductases; and *WSD1*, which encodes a wax ester synthase/diacylglycerol acyltransferase. Furthermore, gene expression analysis of the data in Wos *et al*. (2021) revealed that six cuticle‐related genes were significantly up‐regulated, whereas four genes were down‐regulated in leaves of alpine populations compared to foothill populations (FDR < 0.1), and these differential gene expression patterns were consistently observed when the plants were grown under both the alpine‐like and foothill‐like conditions applied by Wos *et al*., 2021 (Fig. 4b; Table S5).

## Discussion

### Alpine populations have enhanced cuticle‐based protection against water loss

Overall, alpine populations had lower *g*_min_ and WSD values, especially when grown in an alpine environment, consistent with a more efficient protection from water loss after stomatal closure (Körner, 2021). The lower WSD in alpine as compared to foothill populations was additive to the acclimative lowering of WSD in plants grown in the alpine as compared to the foothill common garden, suggesting that a higher acclimation potential for this trait was selected for in the alpine ecotype. By contrast, neither leaf surface structures nor leaf wettability of adaxial and abaxial leaf surfaces differed between ecotypes. However, leaf wettability was plastically adjusted to the growing site with lower leaf wettability in the alpine common garden (Table 1). In *A. arenosa*, leaf wettability is likely independent of trichome density, as foothill individuals have a higher trichome density on both leaf sides than alpine individuals, regardless of growing site (Bertel *et al*., 2022). An adjustment towards lower leaf wettability is likely advantageous in the alpine habitat (Aryal & Neuner, 2010), as the absence of water droplets on leaves may be beneficial at freezing temperatures that can occur throughout the growing season in the alpine environment (Neuner, 2014). Such differences in cuticle traits between plants grown in the foothill and alpine common gardens may reflect high phenotypic plasticity in the populations. A high degree of phenotypic plasticity, although inherently costly in stable environments, may be selected for in habitats characterised by highly variable environmental conditions (DeWitt *et al*., 1998) and is often observed in evolutionarily young plant ecotypes (Szukala *et al*., 2022), for which regional differences in the ecotypic differentiation can also occur (Lowry, 2012; Bertel *et al*., 2018). Indeed, although ecotypes are generally characterised by strong and genetically determined parallelism, both neutral and selective processes can lead to significant non‐parallel deviations in certain traits or populations (Stuart *et al*., 2017; Thompson *et al*., 2019). This could explain some regional variation in cuticle traits between populations of the same ecotype.

Lower *g*_min_ values have been associated with adaptation to reduced soil water availability, but this may not explain the lower *g*_min_ in the alpine ecotype, as similar Ellenberg indicator values (Ellenberg, 1992) for soil moisture were reported for natural foothill and alpine *A. arenosa* habitats (Knotek *et al*., 2020). Acclimative lowering of *g*_min_ in response to drought, higher evaporative demand and higher temperatures have been reported for several plant species (Duursma *et al*., 2019). For example, in conifer needles *g*_min_ increases as air temperatures fall with rising elevation, which has been associated not only with reduced evaporative demand but also with insufficient time to complete cuticle maturation at high elevations (Fernández *et al*., 2017). By contrast, in low‐statured alpine plants, canopy warming, which helps to mitigate cold, can increase evaporative demand, and leaf overheating combined with lower air humidity can dramatically increase the vapour pressure deficit (VPD), the driving physical force of transpiration (Smith & Geller, 1979; Körner *et al*., 1983; Schulze *et al*., 1985; Körner, 2021). In addition, wind can drive evaporation by disturbing the moist boundary layer on leaf surfaces and replacing it with dry air from the surroundings, leading to a steeper moisture gradient between the mesophyll and leaf surface (Körner, 2021). Wind increases VPD and has been reported to induce immediate stomatal closure in many alpine species (Körner & Mayr, 1981). Both increased VPD due to frequent strong winds and canopy‐warming force stomata to close and are typical alpine environmental conditions that are likely driving forces for the heritable formation of a less permeable cuticle in alpine ecotypes.

### Does differentiation in cuticular wax composition account for enhanced protection against water loss?

Cuticle permeability is largely determined by the chemical composition of the cuticular waxes, rather than their total amount within the cuticle, or cuticle thickness (Riederer & Schreiber, 2001). For example, the accumulation of alkanes with longer carbon chain length, which are more hydrophobic, under water stress, resulted in better protection against water loss in *Nicotiana benthamiana* (Asadyar *et al*., 2024).

The leaf cuticular wax constituents identified in *A. arenosa* were similar to those found in *A. thaliana* (Jenks *et al*., 1995) and *A. halleri* (Yumoto *et al*., 2021), with C29, C31 and C33 alkanes being the main components. In *A. halleri*, a higher water repellency of cauline leaves in subalpine, as compared to low‐elevation populations, was associated with a heritably higher accumulation of these alkanes in cuticular waxes, highlighting the potential importance of cuticle composition in the adaptation to growth at different elevations (Yumoto *et al*., 2021). Rosette leaves of subalpine plants also showed higher amounts of the primary alcohols 1‐hexacosanol and 1‐octacosanol when grown in their native environment as compared to low‐elevation individuals, but this difference was not observed in plants grown under the same artificial conditions, suggesting that it resulted from a differential acclimation potential (Yumoto *et al*., 2021). In *A. arenosa*, a clear ecotypic differentiation was also observed for the fatty alcohols, 1‐hexacosanol and 1‐octacosanol, in rosette leaves, with higher contents in alpine than in foothill populations (Fig. 3; Table S6). As a negative correlation between the amounts of very long‐chain primary alcohols and residual transpiration in barley leaves was previously reported (Hasanuzzaman *et al*., 2017), this difference may partly explain the lower *g*_min_ and WSD observed in the alpine ecotype. Furthermore, while no consistent differences between ecotypes were found for the predominant straight‐chain alkanes (C29, C31 and C33), two branched‐chain alkanes, identified as *iso‐*alkanes with 29 and 31 total carbons, were found to be accumulated in the leaf wax of alpine as compared to foothill populations. The presence of branched alkanes in waxes was previously reported in *A. thaliana* and could influence the physical properties of the cuticle (Busta & Jetter, 2017). Indeed, an increase in branched alkanes in response to UV‐B exposure was associated with higher leaf wettability in *Nicotiana tabacum* (Barnes *et al*., 1996).

The overall lower *g*_min_ and WSD in the alpine ecotype together with the ecotypic differentiation in cuticular wax composition may reflect features of the alpine habitat, providing protection against environmental factors such as higher VPD, UV radiation and freezing stress. The lack of substantial regional effects and the overall differences between alpine and foothill populations in common gardens suggest parallel and heritable adjustments across independently evolved populations. Such observed parallelism in physiological traits, together with higher fitness in the native habitat (Wos *et al*., 2022), suggests that they evolved under natural selection.

### The genetic architecture of the alpine ecotype points to an adaptive value of cuticle traits

The identification of traits likely selected for in alpine environments provided a strong basis for investigating the associated genetic architecture. Genomic analysis of foothill and alpine *A. arenosa* populations identified nine candidate genes associated with cuticular wax metabolism in alpine populations, as indicated by outlier SNPs (Fig. 4a). These SNPs may either affect protein function (non‐synonymous SNPs) or control gene expression (upstream region SNPs). Differential gene expression analysis also revealed significant differences in the expression of multiple genes related to cuticle metabolism between alpine and foothill populations (Fig. 4b).

**Fig. 5:**
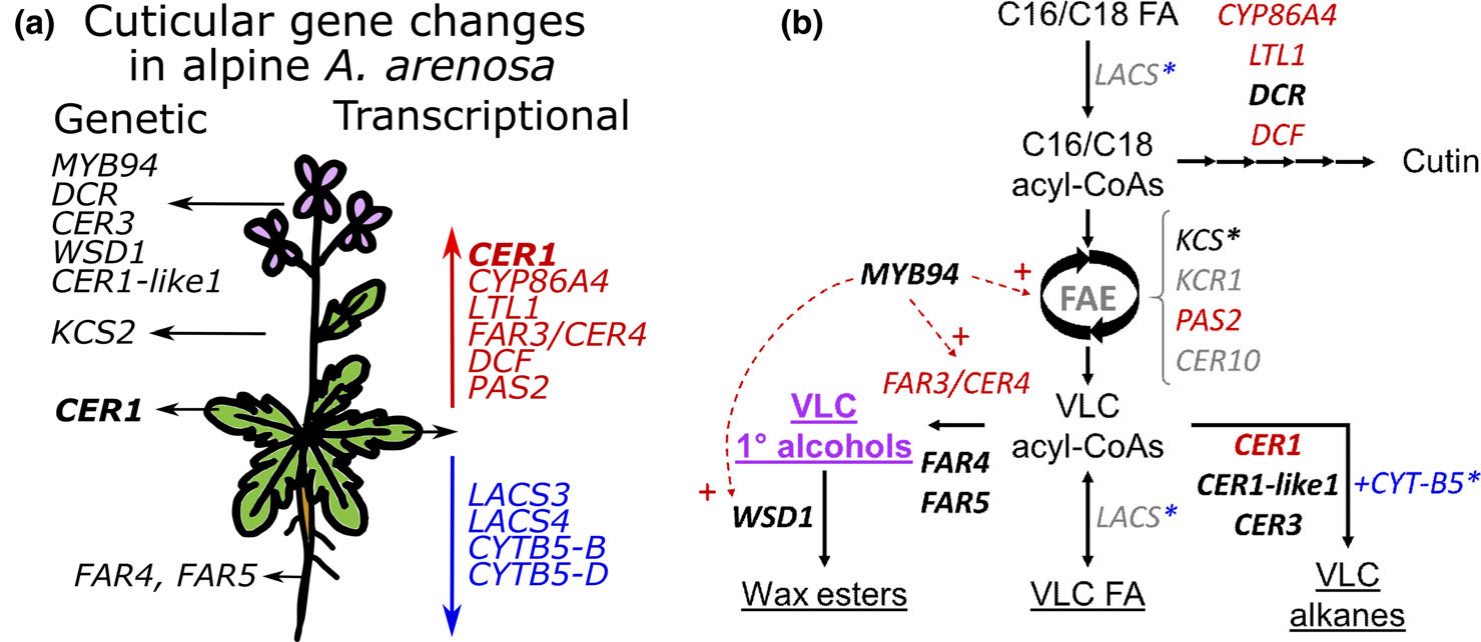
Proposed model for the genetic basis of ecotypic differentiation in cuticular wax metabolism. (a) Summary sketch of genetic and transcriptional differences in cuticle‐associated genes in the alpine *Arabidopsis arenosa* ecotype compared to the foothill ecotype. Black arrows indicate organs where the genes are dominantly expressed in *Arabidopsis thaliana* (Klepikova *et al*., 2016). Red and blue arrows and text indicate up‐ and down‐ regulated genes, respectively. (b) Simplified representation of the biosynthetic pathways for cutin and cuticular waxes, highlighting candidate genes involved. Genes showing outlier differentiation are shown in bold font, up‐regulated genes are shown in red, and down‐regulated genes are displayed in blue. The remaining 86 cuticular genes studied (Supporting Information Table S5), which did not show significant genetic or transcriptomic differentiation, are not shown. Asterisks denote multigenic families, within which certain genes displayed genetic and/or transcriptional variations. Solid black arrows represent enzymatic reactions and dotted red arrows represent transcriptional regulations. FA, fatty acid; FAE, fatty acid elongase complex; VLC, very long chain.

Candidate genes resulting from the selection scans included *CER1*, its genetically linked homolog *CER1‐like1*, and *CER3*, with which they can form a complex catalysing the conversion of acyl‐coenzyme A to alkanes (Bernard *et al*., 2012; Pascal *et al*., 2019). In the case of *CER1*, outlier SNPs were found exclusively in its upstream regulatory region, which may explain why this gene was found up‐regulated in the alpine ecotype, as compared to its foothill counterpart, in the transcriptome data (Fig. 5). Several abiotic stress factors have been shown to influence *CER1* expression in *A. thaliana*, where it was induced by osmotic stress and water deficit, likely through abscisic acid (ABA) signalling (Bourdenx *et al*., 2011). Furthermore, *CER1* overexpression resulted in a dramatic increase in alkane accumulation in the cuticular waxes of aerial organs, in conjunction with a reduced cuticle permeability (Bourdenx *et al*., 2011). However, in *A. arenosa*, the absence of substantial differences between ecotypes for the accumulation of any of the predominant alkanes in the leaf cuticular waxes does not provide further support for a constitutive induction of this pathway in explaining the lower *g*_min_ in alpine populations. Of note, two genes encoding cytochrome B5s, CYTB5‐B and CYTB5‐D, acting as electron donors in the reduction and de‐carbonylation of VLCFA‐CoA to alkanes were found to be down‐regulated in the alpine ecotype (Fig. 5).

Two genetically linked genes, encoding fatty acyl‐coenzyme A reductases, FAR4 and FAR5, which catalyse the conversion of acyl‐coenzyme A to primary fatty alcohols, especially in roots, seed coats and wound‐induced leaf tissue (Domergue *et al*., 2010), also showed clear outlier differentiation between alpine and foothill populations (Fig. 5). However, these two enzymes were shown to have different chain length substrate specificities, and suggested to preferentially catalyse the formation of primary alcohols with 18 and 20 carbons (Domergue *et al*., 2010; Chacón *et al*., 2013). Furthermore, two outlier SNPs were associated with *MYB94*, which encodes a transcription factor involved in the regulation of cuticle biosynthesis, which was shown to activate the expression of *KCS2/DAISY*, *CER2*, *FAR3/CER4*, *CER10* and *WSD1* (Lee & Suh, 2015). FAR3/CER4 was identified as the main fatty acyl‐coenzyme A reductase responsible for fatty alcohol synthesis in the epidermal cell of aerial tissues in *A. thaliana* (Rowland *et al*., 2006). Thus, the up‐regulation of this gene in the leaves of alpine plants as compared to foothill plants could explain the observed higher accumulation of 1‐hexacosanol and 1‐octacosanol (Fig. 3). The accumulation of fatty alcohols, taken together with the genetic polymorphism detected for *WSD1* (Fig. 4), could also indicate a differential biosynthetic regulation of wax esters, the end products of the alcohol‐forming pathway. Indeed, WSD1 catalyses the final step of this pathway, producing esters from fatty alcohols and acyl‐CoA thioesters (Li *et al*., 2008). *WSD1* was previously shown to be induced by drought, salt stress and ABA, leading to wax ester accumulation on *A. thaliana* leaves and stems, whereas the *wsd1* mutant had a reduced wax ester coverage and showed increased leaf water loss (Patwari *et al*., 2019). In maize, the establishment of the water barrier properties in the cuticle of adult maize leaves coincided with the timing of wax ester accumulation, suggesting that they play a prominent role in determining cuticular permeability (Bourgault *et al*., 2020).

In summary, the genetic polymorphism in cuticle‐related genes detected between the alpine and foothill ecotypes could explain their biochemical differentiation and account for some of the observed eco‐physiological adjustments. The ecotypic differentiation in cuticle traits observed in recently evolved populations in the absence of strong regional differentiation, together with the genetic architecture of the alpine ecotype, provides strong evidence for an adaptive value of the cuticle in the colonisation of alpine habitats.

## Author contributions

IK, EA, FK and GN planned and designed the research. DK, JM, EL, CB, WK and EA conducted the fieldwork and experiments. CB, GN, MB, EA, KH, GW and IK analysed and interpreted the data. CB, KH and MB conducted the statistical evaluation. CB, GN, EA and IK drafted the article, which was revised and approved by all authors. CB and EA contributed equally to this work as first authors and GN and IK as senior authors.

## Disclaimer

The New Phytologist Foundation remains neutral with regard to jurisdictional claims in maps and in any institutional affiliations.

## Supporting information


**Dataset S1** Minimum leaf conductance, water saturation deficit, leaf wettability of adaxial and abaxial leaf surfaces and cuticular wax composition of alpine and foothill populations of *Arabidopsis arenosa* grown in common gardens.

**Fig. S1** Geographical localisation of the collection and common garden sites.
**Fig. S2** Surface structures of leaves of the alpine and foothill *Arabidopsis arenosa* ecotypes visualised by scanning electron microscopy.
**Methods S1** Seed collection, regeneration and transplantation experiments.
**Table S1** *Arabidopsis arenosa* populations used in this study.

**Table S2** List of whole‐genome re‐sequenced individuals of *Arabidopsis arenosa* compiled for this study.
**Table S3** Candidate cuticle‐related genes in *Arabidopsis lyrata*.
**Table S4** Candidate SNPs in cuticle‐related genes as identified by allele frequency difference‐based scan.
**Table S5** Differential expression of cuticle genes in *Arabidopsis arenosa*.
**Table S6** Differences in leaf cuticular waxes of alpine and foothill *Arabidopsis arenosa* populations originating from three mountain ranges, grown in an alpine common garden.
Please note: Wiley is not responsible for the content or functionality of any Supporting Information supplied by the authors. Any queries (other than missing material) should be directed to the *New Phytologist* Central Office.

## Data Availability

The data that supports the findings of this study are available in the Supporting Information of this article (Dataset S1) and public repositories (details in Table S2).
