# Supplementary material for: Repeated colonisation of alpine habitats by Arabidopsis arenosa involved parallel adjustments of leaf cuticle traits
Source: New Phytol. 2025 Mar 17;246(4):1597–608. doi: 10.1111/nph.70082 (PMC12018776; doi:10.1111/nph.70082)
Supplement: Supplementary file 2 — Fig. S1 Geographical localisation of the collection and common garden sites. Fig. S2 Surface structures of leaves of the alpine and foothill Arabidopsis arenosa ecotypes visualised by scanning electron microscopy. Methods S1 Seed collection, regeneration and transplantation experiments. Table S1 Arabidopsis arenosa populations used in this study. [file NPH-246-1597-s002.pdf]

New Phytologist **Supporting Information**

Article title: **Repeated colonisation of alpine habitats by *Arabidopsis arenosa* involved parallel adjustments of leaf cuticle traits**

Authors: Clara Bertel, Erwann Arc, Magdalena Bohutínská, Dominik Kaplenig, Julian Maindok, Elisa La Regina, Guillaume Wos, Filip Kolář, Karl Hülber, Werner Kofler, Gilbert Neuner and Ilse Kranner

Article acceptance date: 28 February 2025

The following Supporting Information is available for this article:

Figure S1 Geographical localisations of the collection and common garden sites.

Figure S2 Surface structures of leaves from the alpine and foothill *Arabidopsis arenosa* ecotypes visualized by scanning electron microscopy.

Table S1 *Arabidopsis arenosa* populations used in this study.

Table S2 List of whole genome resequenced individuals of *Arabidopsis arenosa* compiled for this study.

Table S3 Candidate cuticle-related genes in *Arabidopsis lyrata*.

Table S4 Candidate SNPs in cuticle-related genes as identified by using the allele frequency difference-based scan.

Table S5 Differential expression of cuticle-related genes in *Arabidopsis arenosa.*

Table S6 Differences in leaf cuticular waxes of alpine and foothill *Arabidopsis arenosa* populations originating from three mountain ranges, grown in an alpine common garden.

Methods S1 Seed collection, regeneration and transplantation experiments

Dataset S1 Minimum leaf conductance, water saturation deficit, leaf wettability of adaxial and abaxial leaf surfaces and cuticular wax composition of alpine and foothill populations of *Arabidopsis arenosa* grown in common gardens

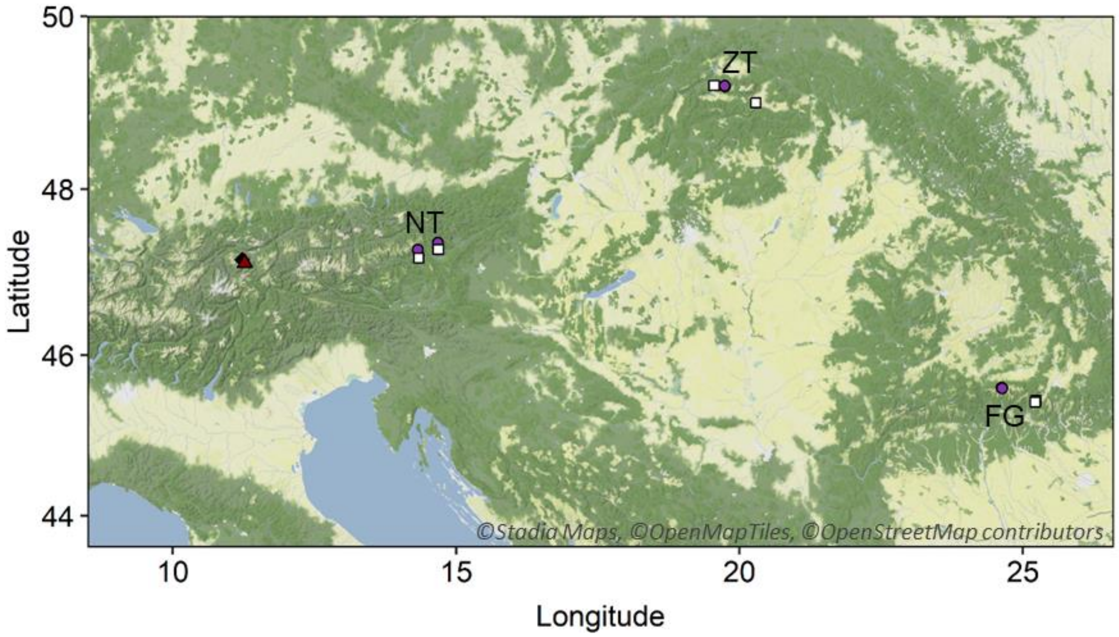

Figure S1 Geographical localisations of the collection and common garden sites. Seed from alpine (purple circles) and foothill (white squares) *Arabidopsis arenosa* populations were collected from three European mountain ranges, the Niedere Tauern (Eastern Alps, Austria, NT), a sub-range of Tatra Mountains [Western Carpathians, Západné Tatry (ZT)] and the Făgăraș mountains (Southern Carpathians, Romania, FG). The experimental alpine (red triangle) and foothill (black diamond) common garden were established in the Alpine Garden on Mount Patsherkofel and in the Botanical Garden of the University of Innsbruck, respectively. This map was generated using the ggmap package in R based on ©Stadia Maps.

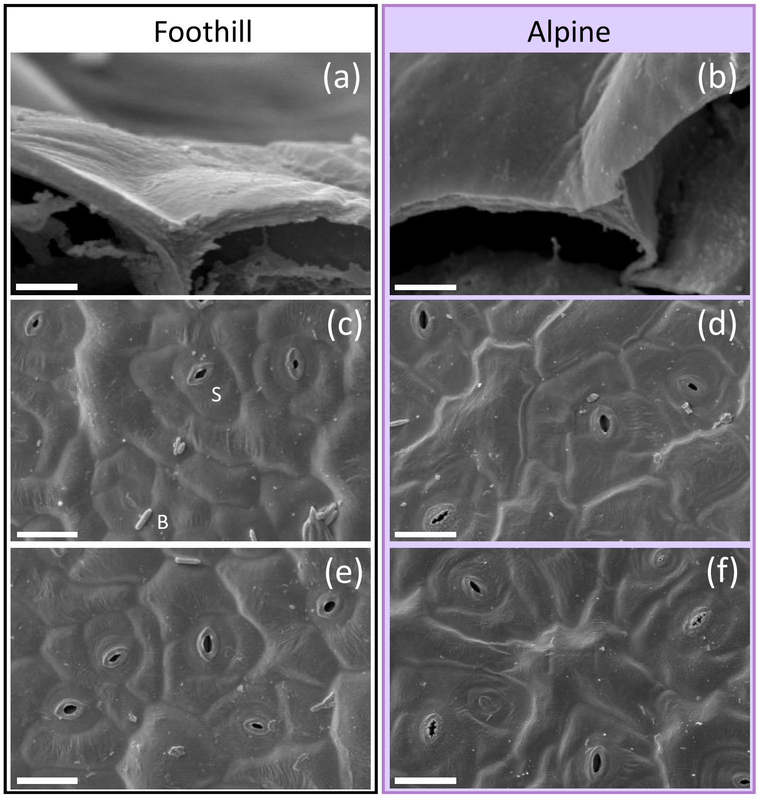

Figure S2. Surface structures of leaves from the alpine and foothill *Arabidopsis arenosa* ecotypes visualized by scanning electron microscopy. Panels (a), (c), and (e) show typical examples of tetraploid populations of the foothill ecotype and (b), (d) and (f) of alpine populations from the Tatra mountains. (a) and (b): cross sections of the epidermis; (c) and (d): adaxial leaf surfaces; and (e) and (f): abaxial leaf surfaces, with stomata embedded in the cuticle. S, Stomata; B, bacteria (occasionally found on across both leaf surfaces); scale bars are (a, b) 2 µm and (c-f) 20 µm.

Table S1. *Arabidopsis arenosa* populations used in this study. The populations described in this study were collected in the field in 2014 as previously described (Wos *et al.*, 2019; Knotek *et al.*, 2020) and have already been used in previous studies (Bohutínská *et al.*, 2021; Wos *et al.*, 2021; Kaplenig et al., 2022; Wos et al., 2022; Bertel et al., 2022). ZT: Západné Tatry, FG: Făgăraș, NT: Niedere Tauern.

Code	Pop.[1]	Lineage	Mountain range	Elevation (m.a.s.l.)	Latitude	Longitude
**Alpine populations**						
**AA087**	-	ZT	Tatra Mountains	2,031	49.197	19.7448
**AA168**	TKO	ZT	Tatra Mountains	1,783	49.2045	19.7352
**AA065**	BAL	FG	Făgăraș Mountains	2,269	45.602	24.6226
**AA222**	LAC	FG	Făgăraș Mountains	2,092	45.5954	24.6346
**AA253**	SCH	NT	Niedere Tauern	2,225	47.2777	14.3219
**AA254**	-	NT	Niedere Tauern	2,360	47.3644	14.6808
**Foothill populations**						
**AA171**	HRA	ZT	Tatra Mountains	720	49.0072	20.2864
**AA229**	-	ZT	Tatra Mountains	673	49.2076	19.5494
**AA067**	DRA	FG	Făgăraș Mountains	858	45.4416	25.2239
**AA251**	-	FG	Făgăraș Mountains	915	45.4267	25.2133
**AA252**	-	NT	Niedere Tauern	820	47.1826	14.3379
**AA255**	ING	NT	Niedere Tauern	970	47.2842	14.6819

[1]*according to Bohutínská et al. (2021)*

Table S6 Differences in leaf cuticular waxes of tetraploid alpine and foothill *Arabidopsis arenosa* populations originating from three mountain ranges, grown in an alpine common garden.

	df	*Sum of Squares*	*F value*	*p-value*
		Hexadecanoic acid | Palmitic acid (FA16:0)		
ecotype	1	0.00007	0.00041	0.9840
mountain range	2	0.43639	1.23047	0.3817
ecotype : mountain range	2	0.02832	0.07986	0.9653
		(9Z,12Z)-Octadeca-9,12-dienoic acid | Linoleic acid (FA18:2)		
ecotype	1	4.47593	13.66691	0.0174*
mountain range	2	0.31293	0.47775	0.6236
ecotype : mountain range	2	0.29215	0.44602	0.9653
		(9Z)-Octadec-9-enoic acid | Oleic acid (FA18:1)		
ecotype	1	0.55180	2.73478	0.3005
mountain range	2	0.34254	0.84884	0.4855
ecotype : mountain range	2	0.17643	0.43720	0.9653
		(9Z,12Z,15Z)-Octadeca-9,12,15-trienoic acid | Linolenic acid (FA18:3)		
ecotype	1	0.61073	0.20832	0.8575
mountain range	2	7.60241	1.29657	0.3749
ecotype : mountain range	2	4.54354	0.77489	0.9653
		Octadecanoic acid | Stearic acid (FA18:0)		
ecotype	1	0.00030	0.00107	0.9840
mountain range	2	0.34147	0.60844	0.5897
ecotype : mountain range	2	0.01986	0.03538	0.9653
		1-Docosanol (C22-OH)		
ecotype	1	0.00297	0.04275	0.9403
mountain range	2	0.08051	0.57992	0.6093
ecotype : mountain range	2	0.11634	0.83801	0.9653
		Heptacosane (C27)		
ecotype	1	0.19735	2.25200	0.4488
mountain range	2	1.06394	6.07032	0.1484
ecotype : mountain range	2	0.18024	1.02837	0.9653
		Octacosane (C28)		
ecotype	1	0.02151	0.23286	0.8575
mountain range	2	0.72055	3.90100	0.1910
ecotype : mountain range	2	0.12180	0.65942	0.9653
		Tetracosanoic acid (FA24:0)		
ecotype	1	0.11055	1.11153	0.6440
mountain range	2	0.72489	3.64435	0.1937
ecotype : mountain range	2	0.08531	0.42888	0.9653
		2-Methyl-octacosane		
ecotype	1	4.82005	38.78371	0.0174*
mountain range	2	1.31133	5.27570	0.1484
ecotype : mountain range	2	0.39583	1.59250	0.9653
		Nonacosane (C29)		
ecotype	1	0.00011	0.00132	0.9840
mountain range	2	0.46031	2.87671	0.2265
ecotype : mountain range	2	0.08811	0.55063	0.9653
		1-Hexacosanol (C26-OH)		
ecotype	1	2.76650	23.89446	0.0196*
mountain range	2	0.87864	3.79443	0.1910
ecotype : mountain range	2	0.01529	0.06602	0.9653

	**2-Methyl-nonacosane**			
ecotype	1	0.43038	5.97066	0.1914
mountain range	2	1.12547	7.80691	0.1456
ecotype : mountain range	2	0.04247	0.29460	0.9653
	**3-Methyl-nonacosane**			
ecotype	1	0.65774	3.42527	0.3005
mountain range	2	0.45722	1.19052	0.4261
ecotype : mountain range	2	0.04031	0.10495	0.9653
	**Triacontane (C30)**			
ecotype	1	0.04257	0.65598	0.7661
mountain range	2	0.62901	4.84677	0.1484
ecotype : mountain range	2	0.03818	0.29416	0.9653
	**Hexacosanoic acid (FA26:0)**			
ecotype	1	0.03756	0.42005	0.8253
mountain range	2	1.51989	8.49859	0.1456
ecotype : mountain range	2	0.10338	0.57805	0.9653
	**1-Heptacosanol (C27-OH)**			
ecotype	1	1.20121	13.00856	0.0527
mountain range	2	1.64155	8.88862	0.1456
ecotype : mountain range	2	0.16099	0.87174	0.9653
	**2-Methyl-triacontane**			
ecotype	1	2.46407	29.91219	0.0174*
mountain range	2	0.85122	5.16661	0.1484
ecotype : mountain range	2	0.33024	2.00444	0.9653
	**Hentriacontane (C31)**			
ecotype	1	0.00505	0.07034	0.9278
mountain range	2	0.73224	5.10029	0.1484
ecotype : mountain range	2	0.02502	0.17425	0.9653
	**1-Octacosanol (C28-OH)**			
ecotype	1	3.53956	21.54054	0.0197*
mountain range	2	0.70001	2.13000	0.2887
ecotype : mountain range	2	0.17855	0.54330	0.9653
	**Cholesterol**			
ecotype	1	0.11708	0.48023	0.8253
mountain range	2	1.27910	2.62322	0.2360
ecotype : mountain range	2	0.72283	1.48240	0.9653
	**Dotriacontane (C32)**			
ecotype	1	0.00985	0.11001	0.9084
mountain range	2	0.95159	5.31414	0.1484
ecotype : mountain range	2	0.02163	0.12080	0.9653
	**Octacosanoic acid (FA28:0)**			
ecotype	1	0.01477	0.12709	0.9084
mountain range	2	1.68725	7.26000	0.1456
ecotype : mountain range	2	0.02889	0.12429	0.9653
	**1-Nonacosanol (C29-OH)**			
ecotype	1	0.07857	0.85455	0.7071
mountain range	2	1.35609	7.37493	0.1456
ecotype : mountain range	2	0.06870	0.37360	0.9653
	**2-Methyl-dotriacontane**			
ecotype	1	0.93620	9.58774	0.0899
mountain range	2	0.56966	2.91695	0.2265
ecotype : mountain range	2	0.21190	1.08504	0.9653
	**Tritriacontane (C33)**			

ecotype	1	0.03074	0.33134	0.8501
mountain range	2	0.48444	2.61048	0.2360
ecotype : mountain range	2	0.01419	0.07647	0.9653
1-Triacontanol (C30-OH)				
ecotype	1	0.44065	4.18309	0.2768
mountain range	2	0.71443	3.39100	0.1974
ecotype : mountain range	2	0.15279	0.72523	0.9653
Triacontanoic acid (FA30:0)				
ecotype	1	0.15076	1.39976	0.5839
mountain range	2	0.36375	1.68864	0.3625
ecotype : mountain range	2	0.18902	0.87747	0.9653
Dotriacontanoic acid (FA32:0)				
ecotype	1	0.24321	1.84288	0.5001
mountain range	2	0.34185	1.29514	0.4130
ecotype : mountain range	2	0.29074	1.10151	0.9653

Type III Analysis of Variance Table obtained by linear mixed models relating ecotype, mountain range of origin (mountain range) and their interaction to each of the twenty-nine wax compounds that were identified. Degrees of freedom are approximated using Satterthwaite's method (Kuznetsova et al. 2017). Nesting of populations within mountain ranges was accounted for by introducing a random factor in the model. Wax compounds were transformed by natural logarithm prior to analysis. P-values were corrected for multiple testing by Benjamini-Hochberg correction.

Methods S1 Seed collection, regeneration and transplantation experiments

Seed collection. Seeds were collected in the field in 2014 from a minimum of 10 mother plants of six alpine and six foothill autotetraploid *Arabidopsis arenosa* populations from three European mountain ranges, the Niedere Tauern (Eastern Alps, Austria, NT), a sub-range of Tatra Mountains [Western Carpathians, Západné Tatry (ZT)] and the Făgăraș mountains (Southern Carpathians, Romania, FG) as previously described (Wos et al., 2019; Knotek et al., 2020). Knotek et al. (2020) showed distinct genetic positions of populations from NT, FG and ZT regardless of the elevation of origin, suggesting that in all of these mountain ranges, an alpine ecotype evolved in parallel from a foothill ecotype. The alpine ecotype is smaller and has less flower stems compared to the foothill ecotype. The alpine ecotype produces pink, and sometimes white flowers, whereas the foothill ecotype produces consistently white flowers (Knotek *et al.*, 2020).

Seed regeneration. In order to minimize maternal effects, a second generation of randomly outcrossed seeds was harvested from plants grown under controlled conditions in growth chambers as described in Wos et al., 2021. The third generation of seeds, used for the subsequently described experiments, was produced in a greenhouse located in the Botanical Garden of the University of Innsbruck as previously described (Bertel *et al.*, 2022).

Transplantation experiments. Leaf minimum conductance (g_{min}) and wettability, and investigations by scanning electron microscopy, were conducted in May and June 2020 (the sampling period was adjusted for each common garden, taking into account the time after snow melt in the alpine common garden) on plants from a reciprocal transplantation experiment carried out from summer 2019 to summer 2021 and comprising a minimum of 40 individuals per population within each common garden as reported in Bertel et al. (2022)

To study the leaf cuticle wax composition, an additional transplantation was carried out in the alpine common garden in 2021. At the beginning of July 2021, after 4 days of cold stratification at 4 °C in the dark, third generation seeds were germinated on water-soaked filter papers (Whatman Grade 1) in Parafilm-sealed petri dishes at 20 °C under constant light. Upon germination, seedlings were transplanted to multipot-trays filled with a mixture of “alpine soil” [consisting of leaf mould, topsoil, lavalit, peat, sand and rock meal (5:2:1:2:2:0.2) used routinely at the Botanical Garden Innsbruck], silicate-sand and vermiculite (8:1:1), and grown in an open

greenhouse under ambient light and temperature conditions. At the beginning of August 2021, 35 to 36 young plants per population (for a total of 576 individuals) were transplanted into individual pots filled with the above-described soil mixture, which were then buried in a sandbed in the alpine common garden and watered as required until sampling at the end of October 2021. For all transplantation experiments, plants were distributed over multiple rows, each containing one individual of each population, randomly ordered within each row. As a result, individuals were distributed over a total plot area of about 3 m².

For our transplantation experiments, cold stratification was systematically used to synchronise seed germination and seedlings were grown at least for the first three weeks under controlled conditions to ensure homogeneous seedling establishment. All seedlings were transplanted at the same developmental stage and at the same time. In the reciprocal transplantation experiment used for most eco-physiological assessments, the seedlings were transplanted in September so that they overwintered in the common garden (ensuring vernalisation – which is required to induce flowering in most alpine populations but not consistently in foothill populations). There was no indication that plants were undergoing senescence at the time assessments were carried out.

Climate data monitoring. Microclimatic data were recorded with climate stations (CR10X and CR1000, Campbell Scientific, Logan, USA) as previously described (Kaplenig et al., 2022; Bertel et al., 2022). Measurements were taken at 5 or 6 min intervals. To measure rosette leaf temperature, Type-T copper-constant thermocouples were placed on the surfaces of at least 10 individuals distributed over the entire plot area. PPFD (photosynthetic photon flux density) was measured with quantum sensors (QS; Delta T-Devices; SKP 215). The daily mean leaf temperature, between the beginning of May (2020-05-10) and the end of June (2020-06-30), period within which most eco-physiological investigations were conducted, was of 8.36 ± 2.92°C (with daily minimum of 3.00 ± 2.56°C and maximum of 17.42 ± 5.25°C) at the alpine common garden, as compared to 17.54 ± 3.08°C (10.92 ± 2.37°C to 32.10 ± 7.31°C) for the foothill common garden. In contrast, the daily maximum irradiation, for the same period, was higher in the alpine common garden (1706.9 ± 544.1 mmol m^{-2} s^{-1}) than in the foothill common garden (1215.7 ± 668.3 mmol m^{-2} s^{-1}).

References

Bertel C, Kaplenig D, Ralser M, Arc E, Kolář F, Wos G, Hülber K, Holzinger A, Kranner I, Neuner G. 2022. Parallel differentiation and plastic adjustment of leaf anatomy in alpine *Arabidopsis arenosa* ecotypes. *Plants* **11**: 2626.

Bohutínská M, Vlček J, Yair S, Laenen B, Konečná V, Fracassetti M, Slotte T, Kolář F. 2021. Genomic basis of parallel adaptation varies with divergence in Arabidopsis and its relatives. *Proceedings of the National Academy of Sciences, USA* **118**: e2022713118.

Kaplenig D, Bertel C, Arc E, Villscheider R, Ralser M, Kolář F, Wos G, Hülber K, Kranner I, Neuner G. 2022. Repeated colonization of alpine habitats by Arabidopsis arenosa viewed through freezing resistance and ice management strategies. *Plant Biology* **24**: 939-949.

Knotek A, Konečná V, Wos G, Požárová D, Šrámková G, Bohutínská M, Zeisek V, Marhold K, Kolář F. 2020. Parallel alpine differentiation in *Arabidopsis arenosa*. *Frontiers in Plant Science* **11**: 561526.

Kuznetsova A, Brockhoff PB, Christensen RH. 2017. lmerTest package: tests in linear mixed effects models. *Journal of Statistical Software* **82**: 1-26.

Rawat V, Abdelsamad A, Pietzenuk B, Seymour DK, Koenig D, Weigel D, Pecinka A, Schneeberger K. 2015. Improving the annotation of *Arabidopsis lyrata u*sing RNA-Seq data. *Plos One* **10**: e0137391.

Wos G, Arc E, Hülber K, Konečná V, Knotek A, Požárová D, Bertel C, Kaplenig D, Mandáková T, Neuner G, et al. 2022. Parallel local adaptation to an alpine environment in *Arabidopsis arenosa*. *Journal of Ecology* **110**: 2448-2461.

Wos G, Bohutínská M, Nosková J, Mandáková T, Kolář F. 2021. Parallelism in gene expression between foothill and alpine ecotypes in *Arabidopsis arenosa*. *Plant Journal* **105**: 1211-1224.

Wos G, Morkovska J, Bohutinska M, Sramkova G, Knotek A, Lucanova M, Spaniel S, Marhold K, Kolar F. 2019. Role of ploidy in colonization of alpine habitats in natural populations of *Arabidopsis arenosa*. *Annals of Botany* **124**: 255-268.
